# Enhancing all-in-one bioreactors by combining interstitial perfusion, electrical stimulation, on-line monitoring and testing within a single chamber for cardiac constructs

**DOI:** 10.1038/s41598-018-35019-w

**Published:** 2018-11-16

**Authors:** Roberta Visone, Giuseppe Talò, Silvia Lopa, Marco Rasponi, Matteo Moretti

**Affiliations:** 1 Department of Electronics, Information and Bioengineering, Politecnico di Milano, Milan, Italy; 2 Cell and Tissue Engineering Laboratory, IRCCS Istituto Ortopedico Galeazzi, Milan, Italy; 3 Regenerative Medicine Technologies Lab, Ente Ospedaliero Cantonale (EOC), Lugano, Switzerland; 4 Cardiocentro Ticino, Lugano, Switzerland

## Abstract

Tissue engineering strategies have been extensively exploited to generate functional cardiac patches. To maintain cardiac functionality *in vitro*, bioreactors have been designed to provide perfusion and electrical stimulation, alone or combined. However, due to several design limitations the integration of optical systems to assess cardiac maturation level is still missing within these platforms. Here we present a bioreactor culture chamber that provides 3D cardiac constructs with a bidirectional interstitial perfusion and biomimetic electrical stimulation, allowing direct cellular optical monitoring and contractility test. The chamber design was optimized through finite element models to house an innovative scaffold anchoring system to hold and to release it for the evaluation of tissue maturation and functionality by contractility tests. Neonatal rat cardiac fibroblasts subjected to a combined perfusion and electrical stimulation showed positive cell viability over time. Neonatal rat cardiomyocytes were successfully monitored for the entire culture period to assess their functionality. The combination of perfusion and electrical stimulation enhanced patch maturation, as evidenced by the higher contractility, the enhanced beating properties and the increased level of cardiac protein expression. This new multifunctional bioreactor provides a relevant biomimetic environment allowing for independently culturing, real-time monitoring and testing up to 18 separated patches.

## Introduction

Myocardial infarction and cardiovascular diseases represent the leading cause of mortality and morbidity worldwide, causing billions of dollars expenses in healthcare systems^1,2^. The poor capability of the heart to regenerate after injuries causes an impairment of functionality, which ultimately provokes the organ failure^3^. In the most severe cases, the ultimate solution is heart transplantation that, however, presents several critical aspects, including the poor availability of organs and the need for immunosuppressive treatments^4^.

For these reasons, great efforts have been made in the field of cardiac tissue engineering (TE) in the attempt to generate functional cardiac constructs that can be used as implantable tissue substitutes or as *in vitro* models for developmental biology, safety pharmacology and drug discovery^3,5,6^. With the final goal of generating *in vitro* constructs with key features of the native myocardium, such as high cellular density and synchronous contraction, different strategies have been proposed. In particular, different TE bioreactors have been designed to recreate a cardiac-specific biomimetic environment^7^, by providing biochemical^8^, mechanical^9–11^ or electrical stimuli^12–14^ and enhancing nutrient transport^8,15–17^.

Bioreactors designed to deliver electrical signals mimicking the native heart electrical activity have been exploited to generate beating and functional cardiac tissue patches. The application of a biphasic electric field characteristic of native heart^18^ allowed the development of constructs showing better conductive and contractile properties with respect to non-stimulated constructs^13,19^. However, in most cases these systems are static culture chambers provided with electrodes^20,21^, which allow the generation of cardiac constructs with limited size. The lack of perfusion impairs the maintenance of appropriate levels of nutrients and oxygen in the inner part of large constructs, resulting in the formation of a necrotic core. Proper culture conditions are found only within the first 100–200 µm from the scaffold surface^15,22,23^, because of the limited penetration depth of oxygen and nutrients driven only by diffusion. This problem is of great impact for cardiac constructs since cardiomyocytes have high metabolic activity and are seeded at high density.

To overcome this limitation and to generate cardiac constructs with a clinically relevant size, different bioreactors allowing the perfusion of culture medium have been designed^15–17^. By means of convective transport, the control over oxygen, nutrients and metabolite levels increases and the generation of uniform engineered cardiac tissues can be achieved. Direct interstitial perfusion has been shown to improve the cellular distribution and viability, enhancing the synchronous contraction properties of the constructs in response to electrical stimulation^15,17^. Furthermore, a slow bi-directional interstitial flow has been demonstrated to be beneficial for the structural, molecular, and electrophysiological properties of the cardiac constructs, improving spontaneous contractility of the engineered tissues^8^.

Even if promising, only few bioreactors coupled the beneficial effect of electrical stimuli with the advantages of interstitial perfusion^24,25^. Indeed, the integration of electrodes into perfused culture chambers implies strict constraints in the design of the systems. Furthermore, within these bioreactors the efficient evaluation of tissue maturation and functionality during culture, by means of optical system (e.g. microscope monitoring), results often impaired. Developing culture systems encompassing all these features can positively impact current challenges in developmental biology studies in the cardiac TE field, such as achieving and monitoring the maturation of the engineered tissue^26^.

In the present study, we developed a new culture chamber conceived to culture 3D cardiac constructs within a controlled biomimetic environment. The developed chamber (i) integrates two electrodes for the electrical stimulation of 3D cardiac constructs; (ii) fits in an incubator-compatible oscillating perfusion bioreactor (OPB) providing the constructs with a bidirectional interstitial perfusion; and (iii) allows the optical assessment of tissue functionality during culture. The chamber design was optimized by means of numerical modeling so to embed an innovative anchoring system to hold the scaffold during culture and to release it for the evaluation of tissue maturation and functionality by contractility tests. Furthermore, we investigated the effects of a biomimetic electrical stimulation coupled with bidirectional perfusion on cultured cardiac constructs, by exploiting the independence of the chambers that can be singularly mounted in the OPB frame and individually assessed.

## Results

### Bioreactor chamber for culturing and assessing cardiac constructs

**Figure 1:**
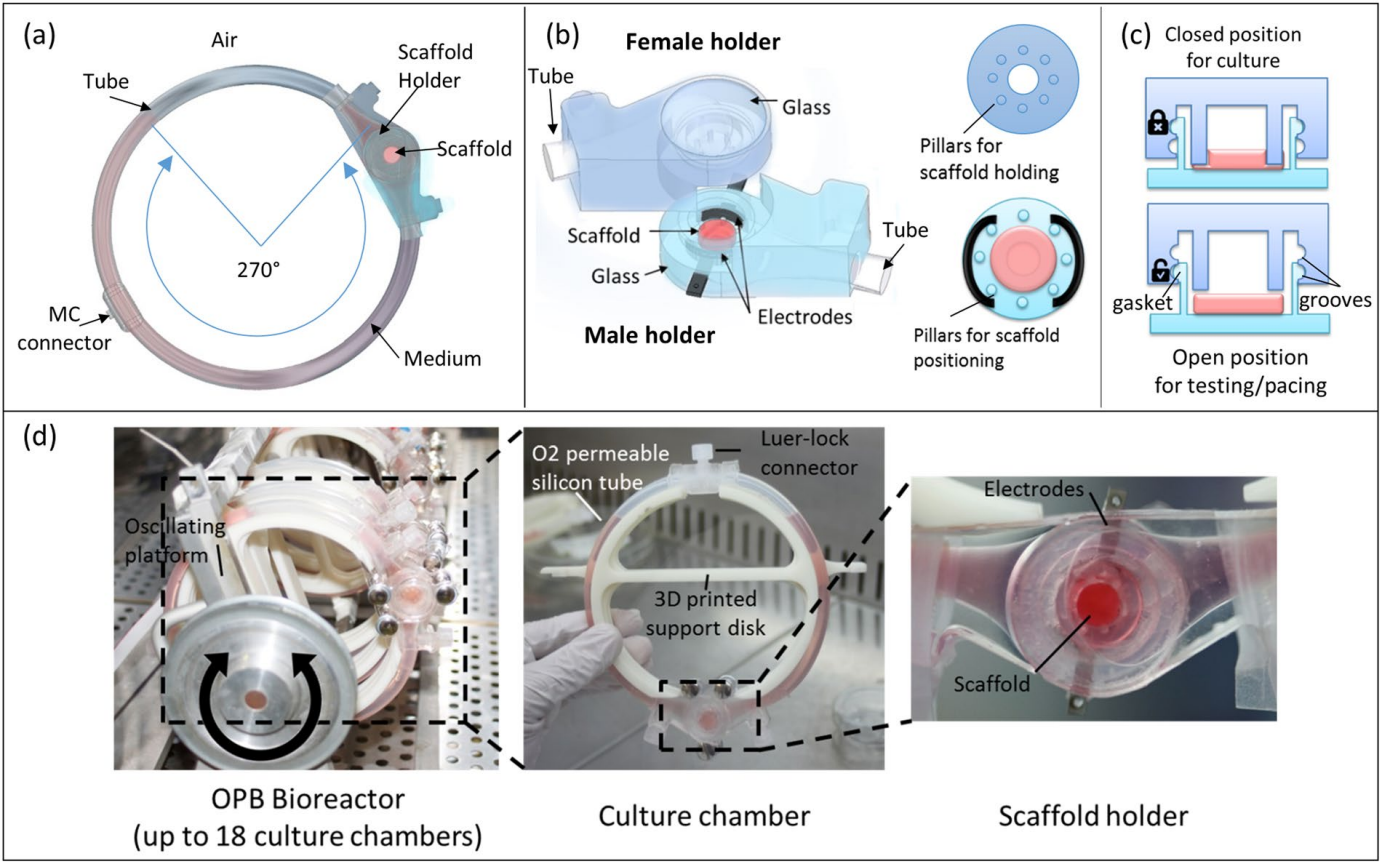
(**a**) Schematic representation of the bioreactor chamber including the scaffold holder and the MC connector linked by a silicone tube; medium perfusion through the scaffold is achieved by bidirectional oscillation (270° spanned angle) along the axis perpendicular to the culture chamber. (**b**) Detailed scheme of the scaffold holder composed by two complementary parts housing electrodes, two round arrays of pillars and glass windows. (**c**) Representation of the two functioning positions of the scaffold holder to hold the construct during culture (top) and to release it for testing (bottom). (**d**) The OPB bioreactor housing the PDMS culture chambers mounted on 3D printed supporting disks; the newly designed chamber allows the optical assessment of constructs during perfusion and electrical stimulation.

Our novel culture chamber was designed to meet three main requirements: (i) providing cardiac constructs with a biomimetic electrical stimulation, (ii) enhancing nutrient exchange through bidirectional perfusion, and (iii) allowing for the assessment of the engineered tissue maturation without interrupting the culture. The chamber design comprises two elements connected through an oxygenating platinum cured silicone tube so to form a toroid: the scaffold holder and a connector for the medium change (Fig. 1a). The closed-loop chamber was mounted on an oscillating platform, which moved bidirectionally along an axis perpendicular to the loop, spanning an angle of 270° and producing medium convection through the scaffold. The scaffold holder was composed by complementary male and female holders, that can be closed by means of a dual-locking system, consisting of a gasket seating into a groove, to house and anchor the construct in a predetermined position (Fig. 1b). The male holder housed two electrodes, the gasket and a circular array of 6 pillars (ø 1.2 mm, h 1.5 mm), to precisely center the scaffold in the culture chamber. The female holder included a circular array of 6 pillars (ø 1.2 mm, h 3 mm) concentric to the male one, providing the mechanical support to withhold the construct, and two grooves to accommodate the gasket in two specific positions (Fig. 1c). As evidenced in Fig. 1c, the two arrays of pillars allowed to hold the scaffold during culture (bidirectional perfusion and electrical stimulation) and to release it during testing (electrical pacing), simply changing the gasket positioning. The culture chamber was completely transparent: the structural parts were realized in polydimethylsiloxane (PDMS), while two glass windows were integrated for high definition imaging (Fig. 1d). The two electrodes were realized in AISI 316L stainless steel and positioned directly during the fabrication process. The connector for medium changes was also realized in PDMS to seal a luer lock connector within the tube. The compatibility with a previously developed OPB^8^ was guaranteed through 3D printed supporting disks connected to the oscillating platform through two magnetic connectors. Figure 1d shows the complete bioreactor supporting multiple culture chambers to independently culture up to 18 constructs in parallel.

### Velocity profile and electrical analyses

**Figure 2:**
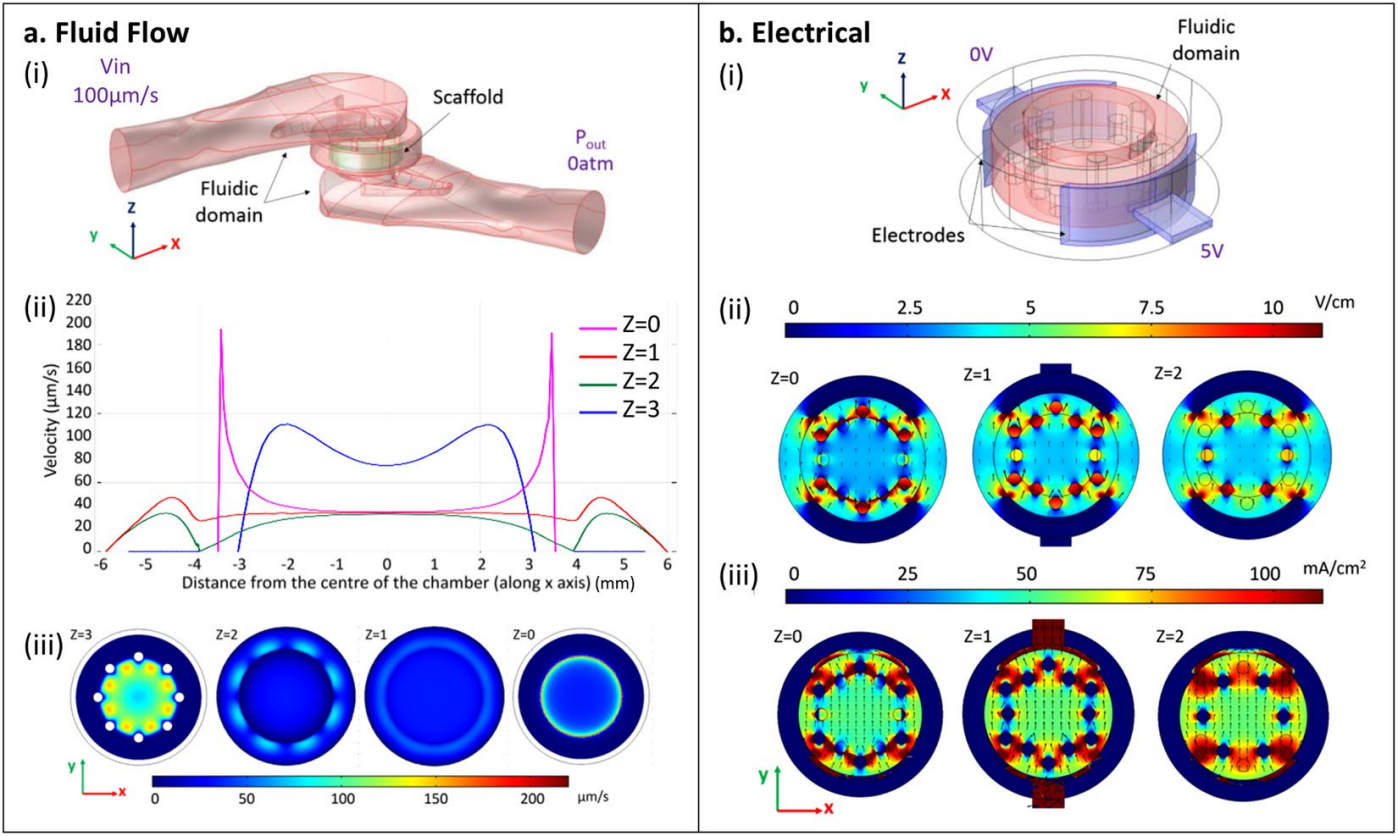
(**a**) Fluid dynamic FEM computational analyses: (i) model representing the medium and the scaffold domains with initial conditions (V = 100 µm/sec; P_out_ = 0 atm); (ii) fluid flow velocity profile evaluated at different quotes of the chamber; (iii) colorimetric maps highlighting the velocity profile at different quotes of the chamber and within the scaffold. (**b**) Electrical FEM computational analyses: (i) model representing the fluidic and the electrode domains with the applied electric field (V = 5 V/cm); colorimetric maps and streamlines (arrows) (ii) of the electric field and (iii) of the current density at different quotes within the chamber.

The internal geometry of the scaffold holder was optimized to achieve a uniform perfusion and a homogeneous electric field within the scaffold. To this aim, fluid dynamic and electrical finite element model (FEM) computational analyses were performed by modeling the culture medium domain with the scaffold (Fig. 2a-i) and the culture medium domain with the electrodes (Fig. 2b-i), respectively.

Fluid dynamic analyses were conducted considering the entire portion of the scaffold holder with the culture medium flowing from the top to the bottom of the scaffold (z = 0 mm) and evaluating the velocity profile across it. The culture chamber (ø 16 mm; −8 mm < r < 8 mm with r = sqrt (x^2^ + y^2^)) and the portion occupied by the scaffold (−4 mm < r < 4 mm; 0 mm < z < 2 mm) spanned from its center (y = x = 0) to the lateral part (r = ±4 mm). FEM studies showed a uniform perfusion speed of about 40 µm/s in the scaffold bulk. Peaks of velocity above 150 µm/s were revealed at the scaffold borders, which are in contact with the scaffold holder (3 mm < x < 4 mm; −3 mm > x > −4 mm; z = 0 mm) (Fig. 2a-ii,iii). Conversely, where the fluid approaches the scaffold (z = 3 mm) the velocity is higher with values ranging between 75 µm/s and 110 µm/s. Lower velocities were found in the center and higher velocities near the array of pillars (Fig. 2a-ii), with peaks of about 150 µm/s between them, as shown by the colorimetric maps (Fig. 2a-iii).

Electrical analyses were conducted by modeling the cylindrical portion of the scaffold holder (ø 16 mm, h 3 mm) and the stainless steel electrodes (h 3 mm) and applying 5 V/cm^13^ as electric field. FEM analyses revealed that the electrodes generated a uniform electric field of 3.5 V/cm throughout the scaffold region (0 mm < z < 2 mm), with peaks between the pillars spanning from 7 V/cm to 10 V/cm. The uniform longitudinal direction of the electric field within the scaffold region was also confirmed by the regular distance and length of the arrows streamlines (Fig. 2b-ii). Similarly, the current density ranged between 50 mA/cm^2^ (z = 0) and 65 mA/cm^2^ (z = 2) in the central part of the chamber and reached 90 mA/cm^2^ between pillars. The distribution of the arrows depicting the current density streamlines confirmed the uniform direction of the current within the construct position (Fig. 2b-iii).

### Electric current measurements

**Figure 3:**
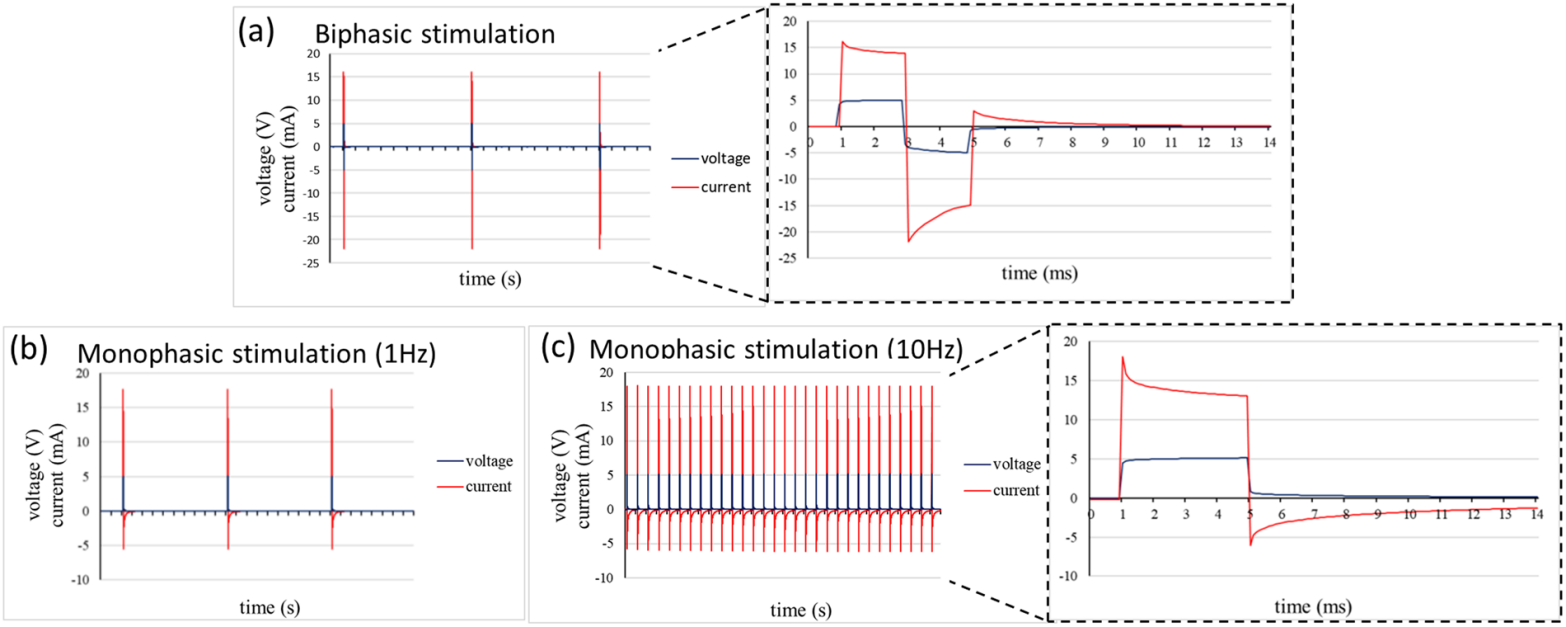
Electric current measurement within the culture chamber when (**a**) a biphasic stimulation of 5 V for 2 ms at 1 Hz, typical for cardiac cell culture is performed within the chamber and when a monophasic stimulation of 5 V for 4 ms at (**b**) 1 Hz or (**c**) 10 Hz, typically used for construct pacing, is provided.

In order to validate the FEM analyses and to verify the suitability of the system to stimulate constructs, the current flux was assessed when a 5 V electric potential was applied. Two patterns were investigated, corresponding to the stimulation during culture (biphasic pulse, 2 ms, 1 Hz) and to the electrical pacing tests (monophasic pulse, 4 ms, 1–10 Hz). As expected, during the biphasic stimulation, the voltage drop across the chamber reached the imposed +5 V and −5 V values with a transient response characteristic of a resistor–capacitor (RC) circuit subjected to a rectangular stimulation (Fig. 3a, blue). The derived values of the characteristic RC time constant (Ƭ) resulted equal to 0.1 ms and 0.3 ms for positive and negative pulses, respectively. The current plot, characterized by an exponential trend (Fig. 3a, red), showed a positive peak of about 16 mA, during the onset of the electrical stimulation, and negative peaks reaching −22 mA when the stimulus inverted the polarity. Positive and negative plateau set at 14 mA and −15 mA, respectively. When the stimulus was switched off, the current rose to 3 mA before dropping to 0 mA after 4 ms. The onset of the current showed a small delay (less than 0.1 ms).

Similarly, during the monophasic stimulation the voltage drop across the chamber reached 5 V showing a characteristic \ of 0.1 ms for both 1 Hz and 10 Hz stimulations (Fig. 3b,c, blue), confirming the suitability of the platform to provide stimulation patterns with frequencies compatible with the testing procedure. The current showed a positive peak of 18 mA, corresponding to the onset of the electrical stimulation, and a negative peak reaching −6 mA when the stimulus was turned off (Fig. 3b, red). The plateau value was around 13 mA, while the time to completely reach the null current in the system resulted equal to 80 ms.

### Optical monitoring of cardiac constructs

**Figure 4:**
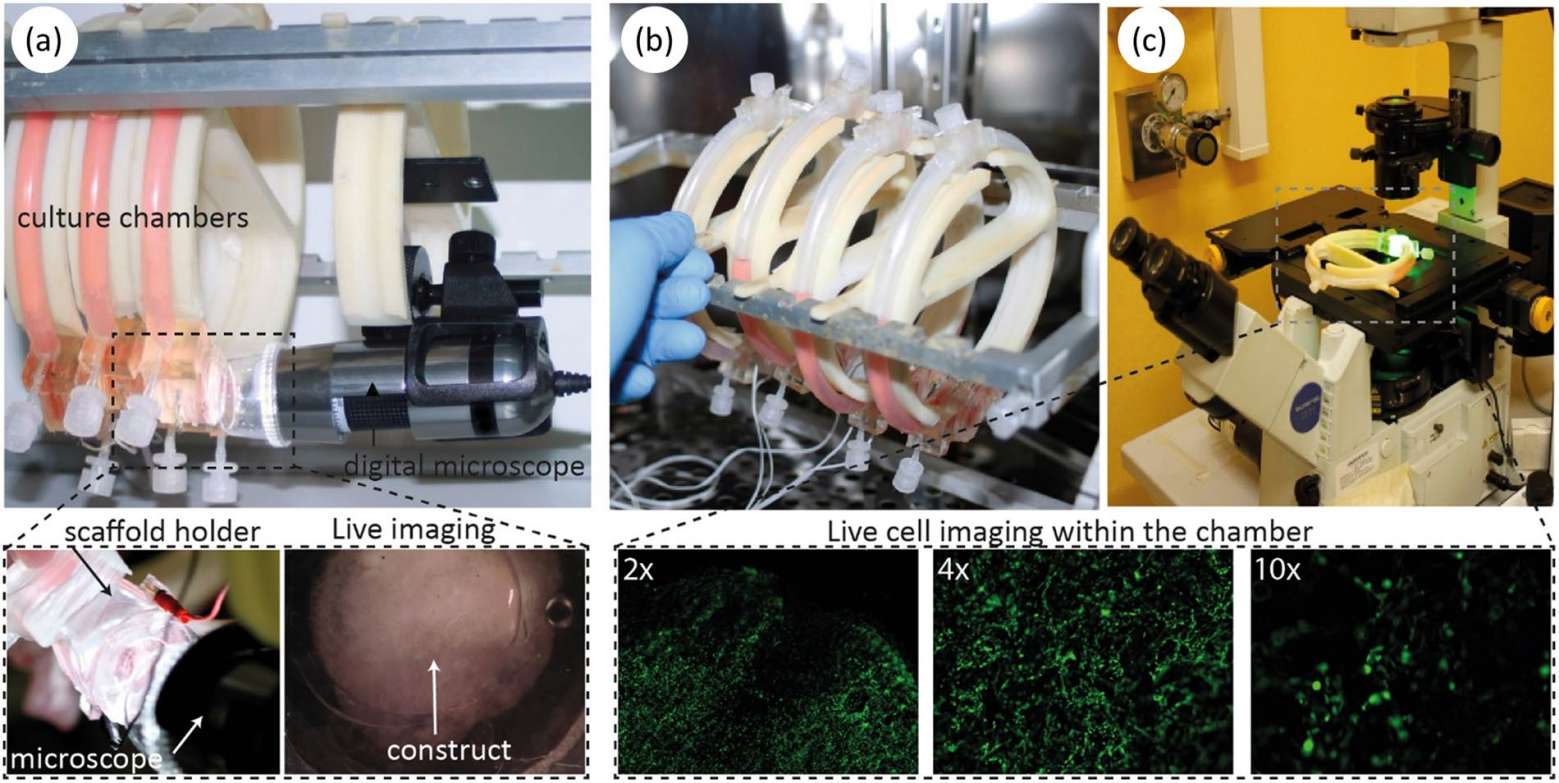
(**a**) The OPB bioreactor housing the transparent culture chambers mounted on 3D printed support disks and integrating a digital microscope for the on-line monitoring of the constructs through the glass windows. (**b**) The OPB allows the release of a single chamber from the bioreactor frame. (**c**) Thanks to the presence of the two transparent glass windows, the construct can be observed using a fluorescence microscope for live imaging of labeled cells without removing the construct from the scaffold holder.

To verify that the design and the optical transparency of the chamber were adequate to real-time assess the cardiac constructs during culture, the scaffolds were monitored using different microscopes. A digital microscope mounted on the OPB was used to monitor cardiac constructs with neonatal rat cardiomyocytes (NRCMs) for the entire culture period. The two glass windows allowed for the on-line imaging of the constructs housed in the scaffold holder (Fig. 4a), providing information about the culture (i.e. scaffold position, air bubbles presence, electrode integrity) and construct functionality (i.e. spontaneous beating, Video S1). Furthermore, to monitor patches at cellular level, the constructs were assessed using a fluorescence microscope by exploiting the single chamber releasing mechanism of the OPB. NRCMs labeled with calcein fluorescent dye were monitored at day 7 during spontaneous beating (Video S2). Cell distribution, morphology, viability and construct functionality (i.e. spontaneous\induced beating) were assessed at different magnifications (Fig. 4b). The onset and the progression of cardiac tissue beating was successfully monitored from day 4 to day 7 without interrupting the culture, observing an initial irregular and fibrillary beating that synchronized and enhanced its strength with time (Video S3).

### Cell seeding and viability

**Figure 5:**
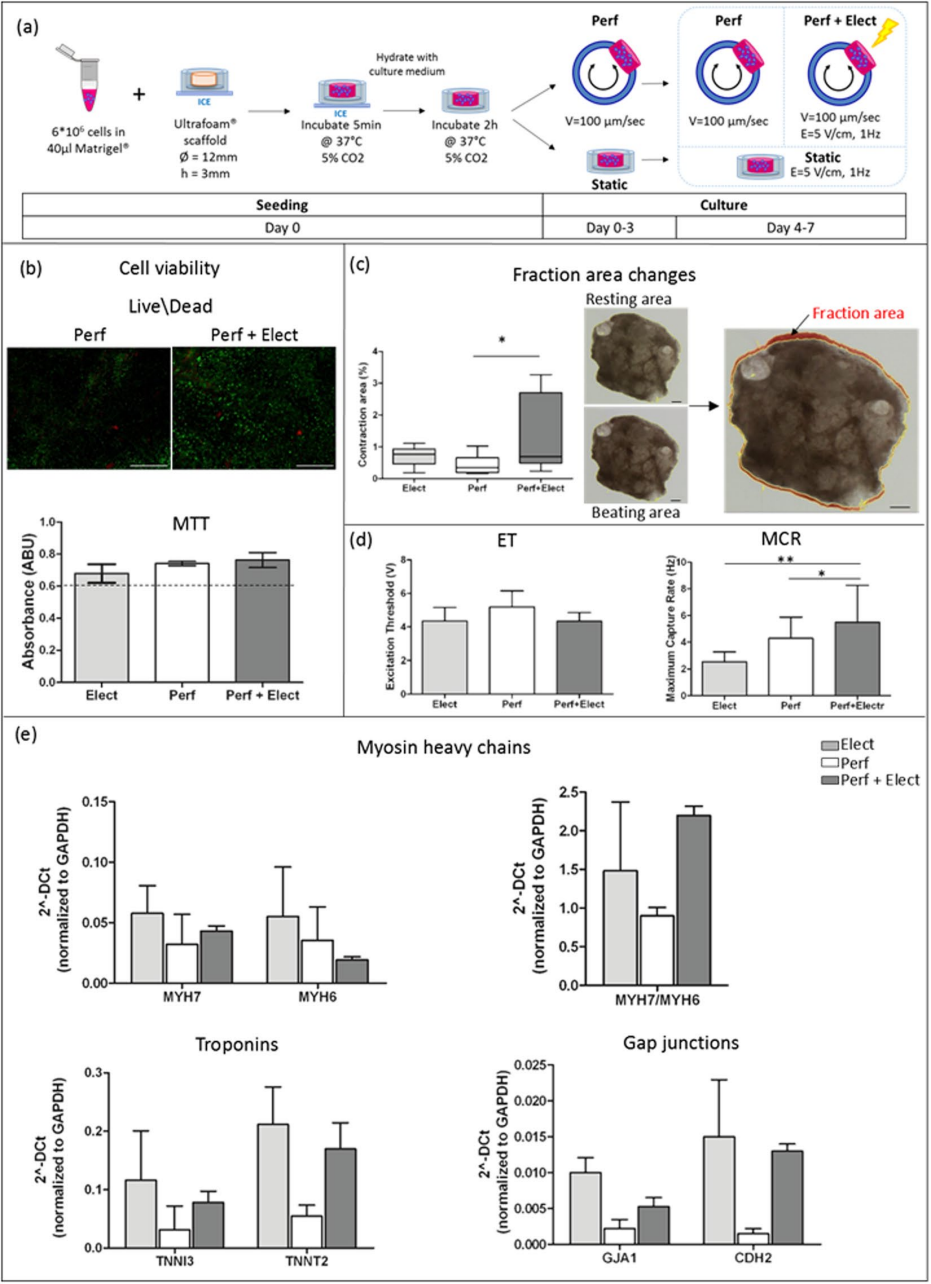
(**a**) Experimental plan: cells were embedded in Matrigel, seeded on a collagen scaffold, let adhere for two hours and placed in the culture chambers; constructs were electrically stimulated for 4 days after a static culture of 3 days (Elect), bidirectionally perfused for 7 days (Perf) or perfused for 7 days in combination with an electrical stimulation, which started after 3 days (Perf + Elect). (**b**) Cytocompatibility of the stimuli provided by the chamber was assessed on NRCF constructs by Live/Dead (scale bars: 500 µm) and MTT assays performed after 7 days of culture. Cardiac functionality was evaluated by: (**c**) measuring the percentage of fraction area change of the construct during its beating (scale bar 1 mm); (**d**) analyzing the ET and MCR parameters during the pacing test; (**e**) evaluating the transcriptional expression of cardiac markers, such as myosin heavy chains, troponins and gap junction proteins, in constructs cultured for 7 days.

To verify the cytocompatibility of the chamber and the provided stimulations, cell viability assays were performed on neonatal rat cardiac fibroblasts (NRCFs) after 7 days in culture. NRCFs embedded in Matrigel were seeded on collagen scaffolds (ø 8 mm, h 2 mm) and let adhere for 2 hours before transferring the construct to the culture chamber. Constructs were provided either with electrical stimulations in static condition (Elect), with bidirectional perfusion (Perf), or with bidirectional perfusion combined with electrical stimulation (Perf + Electr). In both cases (Elect or Perf + Elect), the electrical stimulation was started after 3 days in culture, while the perfusion was set up after 4 hours from the seeding (Fig. 5a). The Live/Dead assay revealed a high NRCF viability over time in all conditions, with cells homogeneously distributed in the scaffold (Fig. 5b). Moreover, the MTT assay on NRCFs cultured for 7 days evidenced a higher metabolic cellular activity, of 13%, 23.5% and 27% for Elect, Perf and Perf + Elect group respectively, as compared to the initial metabolic activity measured 4 hours after seeding (Fig. 5b).

### Assessment of cardiac construct functionality

The positive effects of the stimuli provided were assessed by analyzing the functionality of cardiac patches. After 7 days of culture in the bioreactor, the construct maturation was evaluated by assessing construct beating performances and gene expressions of cardiac key markers.

The on-line monitoring of the constructs proved that NRCMs exhibited spontaneous beating, starting to contract after two days in bioreactor (Video S1). As revealed by immunostaining for cardiac Troponin I (SI 4), cardiomyocytes maintained a higher cell density in all conditions, while showed a more elongated morphology in the electrical stimulated (Elect) and perfused (Perf) group. Electrical stimulation together with bidirectional perfusion enhanced the contraction area percentage reached by the engineered patches. The Perf + Elec group showed a statistically higher contraction distribution (0.69% median, 0.61% Q_1_, 2% Q_2_, 0.23% min, 3.27% max) when compared to Perf condition (0.35% median, 0.21% Q_1_, 0.5% Q_2_, 0.16% min, 1.03% max); while it showed higher values, not statistically significant, respect to the Elect condition (0.76% median, 0.70% Q1, 0.71% Q2, 0.19% min, 0.94% max) (Fig. 5c).

Moreover, concerning functional parameters, the electrical stimulation decreased the excitation threshold (ET), the minimum required voltage to induce a contraction of the cardiac constructs, both when applied alone (Elect) (4.35 ± 0.8 V) or in combination with the perfusion (Elect + Perf) (4.2 ± 0.5 V), respect to the Perf group (5.2 ± 0.95 V) (Fig. 5d). Conversely, the maximum capture rate (MCR), the maximum frequency at which the patch was paced, was significantly higher for the Perf + Elect constructs (5.9 ± 3 Hz) both in comparison to Elect (2.5 ± 0.73 Hz) and Perf group (4.3 ± 1.6 Hz).”

Within the newly developed chamber, the effects of the electrical stimulation, the bidirectional perfusion or the combination of these stimuli were analyzed by assessing the transcriptional expression of cardiac markers. Cardiac maturation was evaluated by investigating the myosin heavy chain-β (MYH7) and -α (MYH6), two isoforms of muscle thick filaments, and assessing their ratio. The capability of developing contraction was evaluated by quantifying the cardiac troponin-I (TNNI3) and troponin-T (TNNT2), two components of thin filaments. Connexin-43 (GJA1) and N-cadherin (CDH2), key proteins of functional gap and adherent junctions of intercalated discs, were finally assessed to evaluate cell-cell coupling (Fig. 5e). The expression at mRNA level of MYH6, indicative of less developed cardiac phenotype, decreased only in the Perf + Elect condition, leading to an increased MYH7/MYH6 ratio (2-fold higher respect to Perf group), implying an higher maturation stage achieved by the cardiac patches. Moreover, electrical stimulation enhanced TNNI3 and TNNT2 level both alone or in combination with perfusion. In details both TNNT2 and TNNI3 resulted 4- and 3-fold higher respectively in the Elect and in the Perf + Elect conditions, compared to the Perf group. Similar results were observed for proteins contributing to cell-cell junction. Levels of GJA1 were 5- and 2.5-fold higher in the Elect and Perf + Elect condition, compared to the Perf group. Moreover, electrical stimulation resulted in level of CDH2 10-fold higher respect to the Perf condition.

## Discussion

In this study, we developed a new bioreactor chamber for cardiac TE that allows providing the cells with a combination of bidirectional perfusion and electrical stimulation and monitoring the construct functionality without interrupting the culture. The effects of the electrical stimulation in static condition, of the sole perfusion or of the perfusion combined with electrical stimulation were assessed measuring the contractile properties of NRCM-seeded constructs after 7 days of culture. The aim of the work indeed was to validate our platform as a suitable tool not only to develop cardiac constructs, but also to evaluate their properties over the culture time. Moreover, to better compare our results with previously reported works^8,24,25^, we coherently decided to investigate the effects of the stimulations in the early phase of the patch formation and by exploiting the well characterized neonatal rat cells, seeded at high cell density as previously suggested^8,13^. Isolation of this primary cell population indeed allows to obtain numerous phenotypically stable cells by following well define and reproducible protocols^11^. Similar motivation (i.e. to compare our results and to obtain more consistent results) pushed us to employ porous collagen sponges as scaffold, which poorly mimic the preferential orientation of the fibers within the heart. This scaffold indeed was widely exploited in previous works^8,13^, for its suitability in generating cardiac patches, exploiting its high porosity and chemical\mechanical properties. Moreover, since the collagen scaffolds implied are commercial, they guarantee a better reproducibility between different seeding and experiments.

The new culture chamber exploited the perfusion principle of the OPB^8,27–29^ and displayed an innovative geometry, housing characteristic features for scaffold positioning and anchoring. Two concentric circular arrays of pillars and a gasket were designed to maintain the scaffold fixed during culture and released during pacing tests, to be free to contract. These technical improvements allowed for the first time the creation of a bioreactor chamber that conjugates interstitial perfusion and electrical stimulation with optical transparency. This unprecedented characteristic is fundamental to monitor and test cell viability and tissue functionality over time, allowing the achievement of an advanced culture systems which can be exploited as a tool to engineer constructs with capacity of cardiac regeneration^30^.

Remarkably, the handling of the culture chamber, the cell seeding procedure, and the chamber positioning within the OPB frame remained simple and user-friendly, as in the previous OPB versions. Real-time and non-destructive evaluation of the progression and maturation level of engineered constructs is a clear need in TE^31^, especially considering the development of autologous tissue grafts for clinical use. Differently from standard pharmaceutical products, advanced therapy medicinal products based on autologous cells are manufactured in small-sized patient-specific batches, leading to a scale-out production system and to difficulties in applying a “Quality by Inspection” methodology. Hence, the implementation of enabling tools to monitor tissue development without directly handling the product appears as a crucial requirement for the rational design of novel technological platforms for TE.

In the past years, different bioreactors have been exploited to achieve a higher control over the culture conditions and, hence, to tailor an *in vivo*-like cardiac cellular environment. By providing a biomimetic electrical stimulation (i.e. rectangular electric pulses 2 ms, 5 V/cm, 1 Hz) the cell-cell electrical coupling^12–14^ was increased and by means of interstitial perfusion^8,16,17^ the heart energy requirement and oxygen consumption were supplied^32^. In particular, a bidirectional perfusion of collagen scaffolds (200 µm/sec) has been demonstrated to elicit spontaneous contraction in cardiac grafts^8^, not achievable with unidirectional and higher flows (400–500 µm/sec)^15,33^. In this perspective, we exploited FEM analyses to optimize the scaffold holder geometry for attaining suitable values of perfusion. Fluid dynamic studies demonstrated a uniform perfusion through the scaffold with velocity never exceeding 200 µm/sec^8^, ensuring an homogeneous nutrient and oxygen delivery to the cells. Furthermore, we investigated by electrical FEM analyses the electric field and current density distribution in the new chamber, when applying a biomimetic electric field of 5 V/cm. Indeed, this has been shown to increase cell-coupling, ultrastructural organisation and synchronous cardiac contractions^13^. Interestingly, the overall electric field remained almost uniform (3.5–4 V/cm) in the central part of the scaffold and increased to 5 V/cm at the borders, due to the presence of the pillar arrays. Values never exceeded the 8 V/cm, lower electric field causing cell death^19^, thus proving the ability of the chamber to supply safe electrical stimulation. Similarly, the current density provided ranged between 50 and 65 mA/cm^2^, getting close to values defined as optimal (i.e. 74.4 mA/cm^2^)^24^.

The electrical characterization of the chamber confirmed the FEM results. Our tests showed a mean positive and negative current of 15 mA, achieving a current density of 50 mA/cm^2^ during the biphasic stimulation of the cardiac constructs. The exponential trend of the current was due to the transduction of charges at the electrode-medium interfaces, while the reverse direction after the stimulus and the higher negative picks were probably induced by the stainless steel electrodes. Stainless steel electrodes have been previously shown to be suitable for the cardiac electrical stimulation, although they leave unrecovered more electrical charges respect to carbon rods^21^. Here, we chose to use stainless steel rather than carbon rods for the superior ease to obtain electrodes with a tailor-made shape and low thickness (1 mm), to include them in the chamber during the fabrication process. The efficacy of stainless steel electrodes to provide monophasic stimulation at frequencies up to 10 Hz was also confirmed, allowing the exploitation of the same electrodes to perform cardiac patch maturation tests. Furthermore, the accumulation of potential cytotoxic by-product release from the electrode nearby our constructs is avoided, since the local media change is continuous.

We optimized both the fluid dynamic and electrical features of the culture chamber geometry to combine these stimulations in our bioreactor, overcoming the limitations of systems providing only one stimulus. For instance, previously designed bioreactors consisting of two carbon rod electrodes fitted in a Petri dish were suitable to generate functional cardiac patches, but failed to maintain a viable tissue beyond 100 µm of thickness due to the limited diffusion of nutrients and oxygen^13,19^. Conversely, perfusion bioreactors providing only interstitial perfusion, enhanced the molecule transport but resulted in cardiac tissue with restricted contractile properties, since a biomimetic electrical stimulation was not provided^8,15–17^. To overcome these limitations, Barash *et al*.^24^ and Maidhof *et al*.^25^ developed two different bioreactors allowing the simultaneous unidirectional interstitial perfusion and electrical stimulation. In the first case, cardiac constructs were positioned between two electrodes and were rigidly fixed within the culture chamber^24^, thus impairing construct contraction and the access for any optical inspection or functional analysis during culture. Despite cells exhibited elongated and striated phenotype and enhanced gap junction expression, the evaluation of cardiac maturation was performed only at the end of the culture by harvesting the patches, and cardiac functionality was not investigated. Maidhof *et al*.^25^ overcame problems related to the rigid positioning of the tissues by holding the scaffolds with a combination of gravity force and downward medium flow, generating engineered patches with enhanced contraction amplitude. However, also in this case, the bioreactor did not allow monitoring and testing the constructs during culture, thus impeding the direct evaluation of the samples characteristics.

Conversely, the design of our bioreactor was intended to perform bidirectional perfusion preventing the rigid fixation of the scaffold, which would impede the contraction of the patch during pacing tests, and the possibility to monitor the construct during culture. The novelty of the culture chamber resides in the new geometry of the scaffold holder introducing two features: the round array of pillars with the gasket and the two glass windows. The PDMS pillars allowed anchoring and maintaining the constructs in place during bidirectional perfusion, while the gasket and the groove provided a dual-locking mechanism to release the scaffold without opening the culture chamber. This allows the construct to fully contract for spontaneous or electrically-induced beating investigation. In addition, the two glass windows allowed each construct to be on-line optically monitored for the entire culture period through integrated digital microscopes to evaluate tissue viability and functionality. These features make the culture chamber suitable for assessing also the effects of drugs by directly adding the compounds within the media, so to monitor any patch alteration on-line or off-line. By envisioning our bioreactor as a platform to perform drug screening, a possible limitation is represented by the scaffold holder, which is made in PDMS. Indeed, even if in our culture chamber the surface to volume ratio is very low (i.e. inner surfaces of the scaffold holder respect to the culture medium volume), PDMS could bind and absorb molecules. For this reason, alternative cytocompatible materials (i.e. derlin, polypropylene and resins) and fabrication techniques (i.e. micromachining, injection molding and 3D printing) could be considered as alternatives to fabricate the scaffold holder and make the platform more suitable for future studies in drug screening.

Other limitations of the previously described bioreactors consist in the need for external peristaltic pumps to move the culture media, increasing the complexity of assembly and handling the culture chambers, which need to be connected to other circuit components (e.g. medium reservoir, pumps, tubes). In our system, the culture chambers are stand-alone and are completely assembled in aseptic conditions before being transferred on the bioreactor frame. Furthermore, previous systems housed more than one patch at the same time^24,25^; while our bioreactor allowed to easily fit up to 18 independent chambers, each one housing a cardiac construct that can be cultured and/or electrically stimulated differently. Each chamber can also be singularly unplugged from the OPB frame to perform temporary tests before continuing culture, to carry out different end-point assays or to harvest a specific construct at a defined time point. This feature results in a significant step-forward both to conduct high-throughput experiments testing multiple culture conditions and to generate patient-specific cardiac patches for clinical use.

In the perfusion conditions, our cardiac constructs exhibited spontaneous beating already after 2 days in culture. The interstitial bidirectional perfusion was started 2 hours after cardiomyocyte seeding, accordingly to Cheng *et al*.^8^; while the electrical stimulation was started after 3 days, to allow cardiac cells to recover and organize contractile proteins and gap junctions^13^. Coherently with other works^24,25^, the electrical stimulation combined with interstitial perfusion significantly enhanced cardiac amplitude contraction, which was up to 3-fold higher respect to the value obtained with the perfusion alone or with the electrical stimulation alone, confirming the superior cellular coupling and contractility achieved. Remarkably, the absolute percentage of the contraction area of our scaffolds in Elect (0,76% median), the Perf (0.35% median) or the Perf + Elect (0.69% median) conditions was higher than the values reached from scaffolds cultured under similar stimulations by Maidhof *et al*.^25^. This superior result was probably achieved thanks to a combination of factors including the scaffold material, the bidirectional fluid flow, and the higher cell density. Interestingly, the higher contractility of the Perf + Elect constructs was confirmed by the lower ET and higher MCR measured, indicating a superior electrical excitability and cellular interconnectivity elicited by the electrical stimulation coupled with perfusion^14^. However, the absolute values of ET resulted higher when compared to the values obtained by Maidhof *et al*. that paced the constructs through carbon rods by applying 2.9 ± 0.4 (electrical no perfused), 2.5 ± 0.5 V (perfused) and 2.7 ± 0.4 V (perfused + electrical)^25^. This was probably related to the different composition of the electrodes, rather than the lower tissue maturation. The carbon rods indeed produce a higher effective field gradient respect to stainless steel electrodes, by retaining 95% of the applied stimulus compared to the 75% of stainless steel^21^. With the exception of the Elect group, comparing the values of MCR of our patches to the results showed by Maidoff (3.5 ± 0.5 Hz Elect, 3.8 ± 0.8 Hz Perf and 4.3 ± 0.6 Hz Perf + Elect), we found that our constructs reached higher frequencies, confirming their functionality.

The positive effect of the electrical stimulation, alone or combined with the perfusion, was further confirmed by the enhanced transcriptional expression of typical cardiac markers. The increased expression of both Troponin-I and Troponin-T indicated a greater organization of the cell sarcomere apparatus when cells are exposed to electrical stimulation^34,35^. The superior levels of Connexin-43 and N-Cadherin detected especially in the Elect and in the Perf + Elect group evidenced a superior electrical cell-cell coupling and a more coordinated cardiomyocytes depolarization^36,37^. However, MYH6 gene resulted slightly downregulated only in the Perf + Elect group, meaning that the coupling of the electrical stimulation with the perfusion (Perf + Elect) led to a more adult-like cardiomyocyte phenotype^38^. This finding is in line with the analyses on cardiac patch functionality, where the Perf + Elect group showed the highest level of contraction during the spontaneous beating and the highest responsiveness to an external electrical pacing. Therefore, electrical stimulation resulted fundamental to increase the electrical properties of the patches, while the perfusion was highlighted to positively affect the overall patch functionality, probably supporting a more uniform distribution of viable cells within the scaffold, that can connect and beat as a syncytium.

In conclusion, we described a novel multifunctional bioreactor chamber that not only performs standard functions (i.e. efficient nutrient supply and waste removal), but also integrates more complex functionalities, specifically tailored to reproduce key features of the cardiac *in vivo* environment (i.e. biophysical stimulation) and to enable a direct and easy evaluation of patch maturation level (i.e. optical monitoring). This TE technological platform was successfully used to generate and culture multiple independent 3D cardiac patches, on which the effects of relevant stimulations (i.e. perfusion and perfusion in combination with biomimetic electrical stimulation) were evaluated by exploiting the optical transparency of the chamber. The innovative features of the proposed bioreactor chamber will be of great interest to generate and analyze functional cardiac patches that can be used as tissue substitute in regenerative medicine applications or as cardiac models in developmental biology and drug discovery, also combining it with more relevant cell sources like human induced pluripotent stem cells-derived cardiomyocytes (hiPSCMs).

## Methods

All reagents for heart dissociation were purchased from Sigma Aldrich (St. Louis, Missouri, USA). Polydimethylsiloxane (Dow Corning Sylgard 184) was purchased from Ellsworth Adhesives. IGF-I was purchased from PeproTech. DMEM, HBSS, primers and probes for rt-PCR from Life Technologies.

### Ethics statement

In this study, cells were isolated from the hearts of 2 day-old neonatal Sprague Dawley rats (Charles River, Wilmington, MA, USA). Animals were involved and euthanized in another study unrelated to this research and approved by the Institutional Animal Care and Use Committee of the San Raffaele Scientific Institute (IACUC 795). All the applicable international, national and/or institutional guidelines for the use of these animals were followed.

### Requirements for the design of the bioreactor chamber

The new bioreactor chamber design was conceived to provide both uniform bidirectional interstitial perfusion and electrical stimulation together with the possibility to optically monitor and assess the patches without interrupting the culture.

The chamber fitted in a previously developed bioreactor, the OPB^8^, consisting of a platform that cyclically oscillates in a pendulum-like motion spanning an angle of 270°. The rotation produces a movement of the culture media within the toroidal culture chamber housed within the bioreactor. This motion determines the interstitial perfusion of the construct contained within a scaffold-holder component, designed to fix the scaffold in a perpendicular position respect to the medium flow direction in the adjacent tubing (Fig. 1a). The scaffold should also be positioned perpendicular to a flat and transparent surface to be optically monitored and accessible for functional tests. Furthermore, to obtain a uniform electric field, the constructs need to be positioned between two electrodes.

### FEM analysis for the scaffold-holder design optimization

To define the most suitable design and configuration of the culture chamber to achieve a uniform fluid flow and a homogeneous electric field, FEM analyses were conducted with a commercially available software (COMSOL Multiphysics 4.2a, Stockholm, Sweden).

Fluid dynamic analysis was performed through the *fluid flow in porous media* interface. The model was composed by the fluidic domain representing the culture medium and the porous domain depicting the construct (ø 8 mm; h 2 mm) (Fig. 2a-i). The medium was considered as an homogeneous, incompressible Newtonian fluid with 1000 kg/m^3^ density and 8.1 × 10^−4^ Pa · s viscosity^39^. The collagen scaffold had a 0.75 porosity and 3 · 10^−11^ k permeability^27^. A tetrahedral mesh with over 3·000·000 elements (60–590 μm) was used. The Navier-Stokes, the Brinkman and the mass continuity equations for incompressible flow fluids were solved. The velocity profile generated within the chamber was studied by setting a normal inflow velocity of 100 µm/sec at the inlet surface and a null pressure at the output (Fig. 2a-i).

The electrical analysis was conducted using the *electric current* interface. The model encompassed the fluidic domain representing the culture medium, the electrode domain depicting the stainless steel electrodes and the culture chamber structures made in PDMS (Fig. 2b-i). The properties of each domain were defined in terms of electrical conductivity (1.5 S/m, 1.32 · 10^6^ S/m and 10^−22^ S/m) and relative permittivity (80.1, 1.005, 2.63) for the culture medium, AISI 316 stainless steel and PDMS, respectively^21,24,40^. A tetrahedral mesh with elements ranging between 500 µm and 5 µm was used. The Maxwell’s equations were solved assuming a steady state conditions with a DC current, as previously reported^21,24^, assuming the PDMS surface as insulating boundary condition and setting the electrodes at ground and 5 V, respectively (Fig. 2b-i). The electric field and current density distribution were assessed.

### Bioreactor chamber design and fabrication

The bioreactor chamber layout and the corresponding molds were designed using a 3D CAD software (Solid Edge ST3, Siemens, Munich, Germany). The scaffold holder and the MC connector were fabricated by replica molding techniques using PDMS and were directly integrated with the silicone tube (1/32“wall Tygon 3350, Cole Parmer, Vernon Hills, IL). Two different molds of PMMA and epoxy resin were assembled to reproduce in negative the features of the male and female parts of the scaffold holder and liquid PDMS was cast into them (10:1 w/w ratio of pre-polymer and curing agent). The geometry of each mold allowed housing a female luer lock (polypropylene, Cole-Parmer, Vernon Hills, IL), while only one mold allowed housing the stainless steel electrodes for their direct embedding within the scaffold holder. After degassing, PDMS was cured at 80 °C for 3 hours. Subsequently, the molds were disassembled and glass windows (ø 22 mm, thickness 1 mm) were glued through silicone. The MC connector mold was fabricated through a 3D printer by fuse deposition modeling.

### Electrical characterization of the chamber

The current flowing through the chamber in response to different electric signals was measured. An electric circuit was set up by placing a 150 ohm resistor in series to the culture chamber and connecting the output channels of a DAQ board (NI USB-6211, National Instrument, TX, USA) to generate the signals. The voltage drop across both elements was directly measured trough the inlet channels of the board, while the current flowing through the circuit was calculated using Ohm’s law. The voltage drop and current through the chamber were assessed when 5 V monophasic (4 ms) or biphasic (2 ms) pulses were applied at 1 Hz and 10 Hz. Results are plotted as means of ten second registration with a sample rate of 10˙000 samples/second.

### Optical systems to monitor constructs

Two different microscopes were exploited for imaging within the chamber. A commercially available digital microscope (Dino-Lite Digital Microscope AM-7013ZT, AnMo Electronics Corporation, Taiwan) was mounted on one of the supporting disks of the bioreactor facing a glass window of a culture chamber, allowing the on-line inspection of the scaffold (Fig. 4a). A fluorescent microscope (Olympus IX-71, Olympus Corporation, Tokyo, Japan) was exploited to off-line assess the constructs within the scaffold holder by removing a single chamber from the OPB frame. The cells, labeled with a fluorescent dye (Calcein AM, Invitrogen Corporation, Isbad, CA, USA) were optically assessed within the culture chambers by 2x, 4x and 10x objectives (PLN, UPL8APO e CPLFLN, Olympus Corporation, Tokyo, Japan).

### Cell isolation

Fibroblasts and cardiomyocytes were isolated from 2 day-old Sprague Dawley rat hearts, following a previously described protocol^16^. Briefly, after heart collection, atria were discarded while ventricles were quartered and digested overnight in 0.06% (w/v) trypsin in Hank’s balanced salt solution (HBSS, Gibco). The day after, the tissues were transferred in 0.1% (w/v) collagenase type II (Worthington Biochemical Corporation, Lakewood, NJ) in HBSS and 5 rounds of digestion for 4 minutes at 37 °C at 100 rpm were performed. To enrich the population for cardiomyocytes, a pre-plating in polystyrene culture flasks was performed for 1 hour in cardiomyocyte complete medium, composed by Dulbecco’s modified essential medium high glucose (DMEM, 4.5 g/l glucose) supplemented with 10% v/v fetal bovine serum (FBS, Hyclone), 100 U/mL penicillin, 100 µg/mL streptomycin and 10 mM hepes. Neonatal rat cardiomyocytes (NRCMs) were used immediately after the pre-plating phase, while neonatal rat cardiac fibroblast (NRCFs) were cultured and used at P3.

### Cell seeding and culture

Scaffolds were obtained by punching 14 mm disks from commercial 3 mm collagen sponge sheets (Avitene Ultrafoam, Davol Inc., Warwick, RI, USA). Once hydrated, the scaffolds showed an average diameter of 8 mm and a height of 2 mm. For cell seeding, each dry scaffold was fitted in a silicon holder (ø 8 mm) and seeded with 6 × 10^6^ cells embedded in 40 µl of 1.2% w/v Matrigel^®^ (Becton-Dickinson, Franklin Lakes, NJ) by uniformly pipetting the solution on the scaffold. After 5 minutes in the incubator, scaffolds were hydrated by adding 40 µl of complete medium every hour. After 2 hours, each construct was transferred in a chamber: scaffold was gently positioned in the male scaffold holder within the largest crown of pillars and the female scaffold holder was closed onto it. The chamber was then housed on the 3D support disk, filled with 10 ml of media from the MC connector and mounted on the OPB frame in the incubator. Instead, electrically stimulated patches in the static condition were positioned in an ad-hoc produced culture chamber composed only by the male part of the scaffold holder, which fitted within a well of a 6-multiwell plate. The volume of the culture media was kept the same as in the bioreactor chambers (i.e. 10 ml). For NRCF and NRCM culture, complete medium or complete medium supplemented with 100 ng/ml of IGF-I were used, respectively.

Constructs were cultured for 7 days within the bioreactor in two experimental conditions: perfusion with a linear flow velocity of 100 µm/sec (Perf) and perfusion coupled with electrical stimulation (Perf + Elect). The electrical stimulation consisted of biphasic rectangular waves at 1 Hz (5 V/cm amplitude and 2 ms duration). Perfusion was started right after all the chambers were positioned within the OPB (2 hours from seeding), while the electrical stimulation was provided by day 3. Medium change was performed after 3 days in culture.

### Cell viability

Cell viability was assessed by 3-(4,5-dimethylthiazol-2-yl)-2,5-diphenyltetrazolium bromide (MTT, Sigma-Aldrich Corporation,) and Live/Dead (Invitrogen Corporation, Isbad, CA, USA) assays on NRCF patches cultured and stimulated for 7 days.

To perform MTT analysis, each sample was extracted from the chamber, washed in PBS and placed in a 24 multi-well plate in the presence of 0.5 mg/ml of MTT in DMEM. After 3 hours of incubation in the dark at 37 °C, the samples were washed in PBS and placed on an agitating plate in a solution of 11.1% (v/v) hydrochloric acid in ethanol for additional 3 hours. The absorbance of the resulting solution was measured at 570 nm using a spectrophotometer (Victor X3; PerkinElmer, Waltham, MA).

The Live\Dead assay was performed according to the manufacturer’s protocol. After washing with PBS, the constructs were incubated with a 2 μM Calcein AM and 4 μM Ethidium homodimer-1 solution for 40 minutes at 37 °C. Labeled cells were imaged using a fluorescence microscope (Olympus IX-71, Olympus Corporation, Tokyo, Japan).

### Functional analyses of cardiac constructs

Cardiac constructs cultured for 7 days were electrically paced through a monophasic square pulse of 4 ms with tunable amplitude (0–10 V) and frequency (1–10 Hz)^19^. The ET was determined by stimulating the constructs with a signal at 1 Hz and measuring the minimum amplitude required to induce synchronous contractions. The MCR was assessed by fixing the signal amplitude (i.e. 1.5-fold the specific ET), and measuring the maximum frequency at which the construct showed a synchronous contraction triggered by the pacing signal. All the measurements were performed maintaining the constructs in complete medium in a 24 multi-well plate between two stainless steel electrodes and observing the samples under a stereomicroscope (Olympus SZX10, Olympus Corporation, Tokyo, Japan). Results were obtained from 8 constructs prepared in 3 independent experiments and are represented as mean ± standard deviation. Statistical analyses were performed using the unpaired t test, after checking the normality of the data.

Fractional area change was evaluated extracting frames from videos recorded during the pacing tests using a digital camera (Digital Sight DSX10, Nikon, Tokyo, Japan) mounted on a stereomicroscope. The frames were analyzed using ImageJ (open source: http://rsbweb.nih.gov/ij) and applying a color threshold to evaluate the entire scaffolds area during rest and at maximum contraction (Fig. 5c). Results were graphed by creating box-and-whisker plots in which the median is the central line of the box that extended from the 25^th^ (Q_1_) to 75th (Q_2_) percentiles and the whiskers represents the range of the measured values. Statistical analyses were conducted using the non-parametric Mann Whitney test (n = 8).

### Fluorescent staining of paraffin-embedded patches

After 7 days of culture scaffold were harvested and fixed for 1 h in 4% paraformaldehyde and then washed 2 times in PBS. Samples were dehydrated through a grade series of ethanol, embedded in paraffin, sectioned (about 5-μm-thick) and mounted on glass slides. Immunofluorescent staining was performed by means of cardiac Troponin I (mouse anti rat, Santa Cruz Biotechnology, 1:100) primary antibody. Mounted samples were permeabilized with 0.1% TritonX-100 for 10 min and treated with 2% bovine serum albumin (BSA, Sigma Aldrich) for 1 h. Primary antibodies diluted in 0.5% w/v BSA were incubated overnight at 4 °C. After washing twice with PBS, secondary antibodies (goat anti-mouse rodamine-conjugated, Thermo Fisher Scientific, 1:200 in 0,5% w/v BSA) were incubated in the dark for 6 hours at 4 °C. DAPI counterstaining was used to identify cell nuclei, by incubating the dye in PBS for 10 minutes at room temperature. Sections were analyzed using a fluorescence microscope (Olympus 517 IX-71).

### Transcriptional expression of cardiac markers

Quantitative real-time reverse transcriptase polymerase chain reaction (RT-PCR) was performed on cardiac constructs after 7 days of culture. For cell harvesting, scaffolds were removed from the chambers, washed twice in PBS and frozen at −80 °C. The day after, each sample was immersed in lysis buffer (PureLink, Life Technologies) with 1% v/v β-mercaptoethanol (Sigma-Aldrich) and constructs were dissolved using a homogenizer (TissueRuptor, QIAGEN). Total RNA extraction was performed using the PureLink RNA Mini Kit (Life Technologies), following the manufacturer’s instructions. Purified RNA was quantified with a spectrophotometer (Nanodrop, Thermo Scientific) and 900 ng of RNA were retrotranscribed to cDNA using the iScript cDNA Synthesis Kit (Bio-Rad Laboratories).

RT-PCR analysis was then performed to evaluate the gene expression of markers for cardiac maturation (MYH7 Rn01488777_g1 and MYH6 Rn00691721_g1), phenotype (TNNI3 Rn00437161_g1 and TNNT2 Rn00562059_m1) and cell-cell coupling (GJA1 Rn01433957_m1 and CDH2 Rn00580099_m1). In details, 50 ng of cDNA were added to a reaction mix composed of 50% v/v TaqMan^®^ Gene Expression Master Mix and 5% v/v TaqMan^®^ Gene expression probes. Duplicates were prepared for each sample and relative expression level of each gene was normalized using the GAPDH gene (glyceraldehyde-3-phosphate dehydrogenase, Rn01775763_g1) and calculated using the 2^−ΔCt^ method (all reagents were from Applied Biosystems).

## Electronic supplementary material

Supplementary Information

Video S1

Video S2

Video S3
