# Supplementary material for: Enhancing all-in-one bioreactors by combining interstitial perfusion, electrical stimulation, on-line monitoring and testing within a single chamber for cardiac constructs
Source: Sci Rep. 2018 Nov 16;8:16944. doi: 10.1038/s41598-018-35019-w (PMC6240103; doi:10.1038/s41598-018-35019-w)
Supplement: Supplementary file 1 — Supplementary Information [file 41598_2018_35019_MOESM1_ESM.docx]

**Supplementary Information**

**Enhancing all-in-one bioreactors by combining interstitial perfusion, electrical stimulation, on-line monitoring and testing within a single chamber for cardiac constructs**

Roberta Visone^1,±^, Giuseppe Talò^2,±^, Silvia Lopa^2^, Marco Rasponi^1^, Matteo Moretti^2,3,4*^

*^1^Department of Electronics, Information and Bioengineering, Politecnico di Milano, Milan, IT;*

*^2^Cell and Tissue Engineering Laboratory, IRCCS Galeazzi Orthopaedic Institute, Milan, IT;*

*^3^Regenerative Medicine Technologies Lab, Ente Ospedaliero Cantonale (EOC), Lugano, Switzerland.*

*^4^ Cardiocentro Ticino, Lugano, Switzerland*

**^*^**corresponding author: [matteo.moretti@grupposandonato.it](mailto:matteo.moretti@grupposandonato.it)

^±^equally contributed

Video SI1: Spontaneous beating of the cardiac patches within the scaffold holder monitored on-line by means of an integrated digital microscope.

Video SI2: Spontaneous beating of the calcein-AM stained cardiac patches within the scaffold holder monitored off-line by means of a fluorescent microscope after 7 days of culture.

Video SI3: Spontaneous beating of the calcein-AM stained cardiac patches monitored off-line day by day (from day 4 to 7) to investigate the onset and progression of the engineered construct beating.


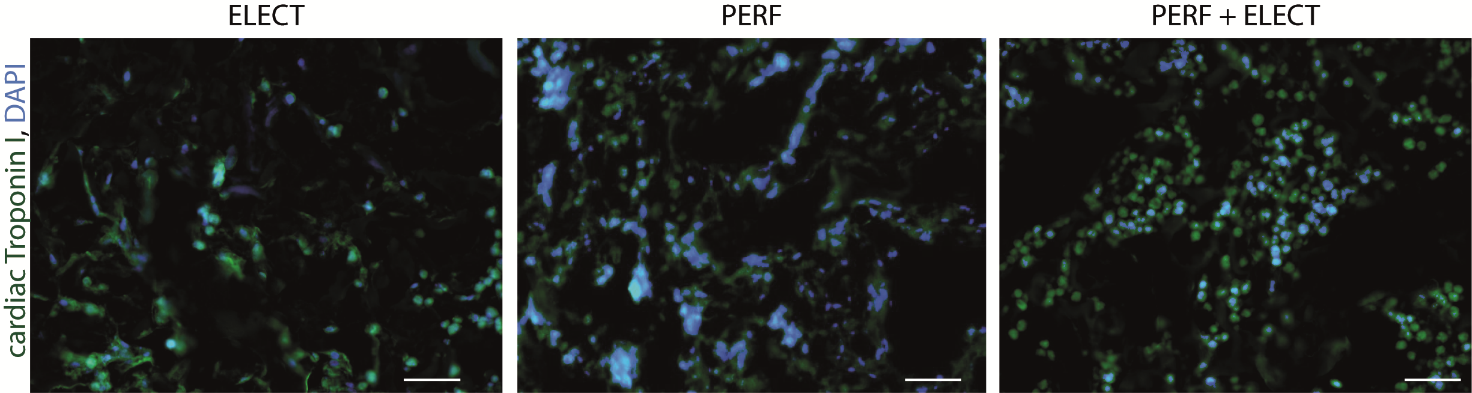


**Figure SI4**: Expression of cardiac Troponin I in the cardiac patches cultured for 7 days within the bioreactor chamber and subjected to the electrical stimulation in static condition (Elect), to the bidirectional perfusion (Perf) and to the combined stimulation (Perf + Elect). Scale bar 100 µm.
